# Everybody wants it done but nobody wants to do it: an exploration of the barrier and enablers of critical components towards creating a clinical pathway for anxiety and depression in cancer

**DOI:** 10.1186/s12913-015-0691-9

**Published:** 2015-01-22

**Authors:** Nicole M Rankin, Phyllis N Butow, Thida Thein, Tracy Robinson, Joanne M Shaw, Melanie A Price, Kerrie Clover, Tim Shaw, Peter Grimison

**Affiliations:** Translational Research Fellow, Sydney Catalyst, The University of Sydney, Chris O’Brien Lifehouse, Level 6, 119-143 Missenden Road, Camperdown, NSW 2050 Australia; Psycho-Oncology Co-operative Research Group, School of Psychology, The University of Sydney, Sydney, Australia; Workforce Education and Development Group, Sydney Medical School, The University of Sydney, Sydney, Australia; Calvary Mater Newcastle Hospital, Newcastle, Australia; Chris O’Brien Lifehouse, Missenden Road, Camperdown, NSW Australia

**Keywords:** Cancer, Oncology, Critical pathway, Barrier analysis, Anxiety, Depression

## Abstract

**Background:**

This study aimed to explore barriers to and enablers for future implementation of a draft clinical pathway for anxiety and depression in cancer patients in the Australian context.

**Methods:**

Health professionals reviewed a draft clinical pathway and participated in qualitative interviews about the delivery of psychosocial care in their setting, individual components of the draft pathway, and barriers and enablers for its future implementation.

**Results:**

Five interrelated themes were identified: ownership; resources and responsibility; education and training; patient reluctance; and integration with health services beyond oncology.

**Conclusions:**

The five themes were perceived as both barriers and enablers and provide a basis for an implementation plan that includes strategies to overcome barriers. The next steps are to design and deliver the clinical pathway with specific implementation strategies that address team ownership, endorsement by leaders, education and training modules designed for health professionals and patients and identify ways to integrate the pathway into existing cancer services.

## Background

High rates of psychological morbidity in cancer patients and their caregivers have been well documented over the past three decades [1-4]. The benefits of routine screening for distress [5,6] in the cancer setting is being debated internationally due to the considerable time and costs associated with such screening [7]. Screening is unlikely to be routinely implemented without compelling evidence that it results in improved patient outcomes.

A review of intervention trials evaluating distress screening reported benefits in 17 of 24 studies; these benefits were primarily restricted to improved communication and referral for psychosocial help with only six of 14 randomised trials reporting benefits to patient well-being [8]. The most significant barrier to screening was the provision of appropriate after-care; only one in three patients received treatment after a positive screen for distress [8]. It appears that in isolation, screening for distress is not sufficient to impact on patient outcomes [9,10] and currently screening is primarily implemented for research purposes [11].

Clinical pathways are used across many health conditions and are one strategy that could improve screening and after-care appropriate to the patient’s anxiety or depression. Pathways have shown success in bringing about change in patient management, depending on context and implementation [12,13]. Relatively few pathways have been developed to deal with psychological distress following cancer diagnosis [14,15] and these do not include explicit strategies or tools to address barriers and encourage uptake. Evidence based interventions for physical symptoms, such as managing pain and breathlessness (dyspnoea) in cancer patients, are also in development [16,17].

Conducting a barrier and enabler analysis is a precursor step to designing implementation strategies [18,19]. Documented barriers and enablers to implementing clinical pathways outside cancer settings [20-22] highlight the lack of: resources; education and training; support from leaders; and patient reluctance to accept help or poor uptake [23]. The most recent Cochrane Review of tailored interventions to overcome identified barriers to changing practice highlights that research is yet to establish the most effective ways to identify barriers, which are the most important to address, or how to select interventions to overcome them [24]. The aim of this paper is to explore the most significant barriers and enablers from the perspective of health care providers for the implementation of a draft clinical pathway for anxiety and depression in cancer patients in the Australian context.

## Methods

### Participants

Medical, nursing and allied health professionals with extensive clinical experience were purposively sampled across multiple disciplines from the membership of the Australian Psycho-Oncology Co-operative Research Group [PoCoG], a national network of clinicians and researchers interested in psycho-oncology.

Potential participants were emailed an invitation and provided with an information statement and consent form [which they were asked to email back] and the draft clinical pathway document. Ethics approval was obtained from the Sydney Local Health District Human Research Ethics Committee [X12-0301].

### Procedure and data collection

A draft evidence-based clinical pathway for managing anxiety and depression in cancer patients tailored to the Australian setting was developed prior to the interviews and is described elsewhere [25]. A semi-structured qualitative interview schedule was developed and was open-ended and exploratory in nature. The interview was designed to seek health professional’s views about the pathway elements, where these professionals represented stakeholder groups who would be future implementers of the pathway in clinical practice. Consenting participants reviewed the draft pathway prior to participating in the semi-structured interview and questions focused on the delivery of psychosocial care in their setting, individual components of the pathway, and barriers and enablers for its implementation. Interviews were conducted face-to-face or by telephone, audio recorded and transcribed verbatim, with recruitment ongoing until no new themes emerged over three consecutive interviews [theoretical saturation].

### Analysis

A thematic analysis was conducted. The first six transcripts were reviewed and discussed by three authors (NR, TR, PB) and a draft coding frame was developed and refined, with differences resolved through consensus. The final coding frame was applied to the remaining transcripts, with super-ordinate themes identified on coding completion. The NVIVO qualitative data program was used for data entry, review and coding purposes.

## Results

The final sample comprised 12 participants from across eight disciplines [Table 1]. Mean interview length was 37.5 minutes [SD 11.3].

**Table 1:** **Sample demographics**

**Professional group**		**N = 12**	**Clinical experience [years]**
Medical	Psychiatrist	2	20, 25
	Medical oncologist	1	5
	Palliative care physician	1	20
	General practitioner	1	25
Allied health & nursing	Psychologist	2	3, 6
	Social worker	2	12, 33
	Oncology nursing	2	23, 25
	Palliative care nursing	1	15

### Thematic analysis

**Figure 1:**
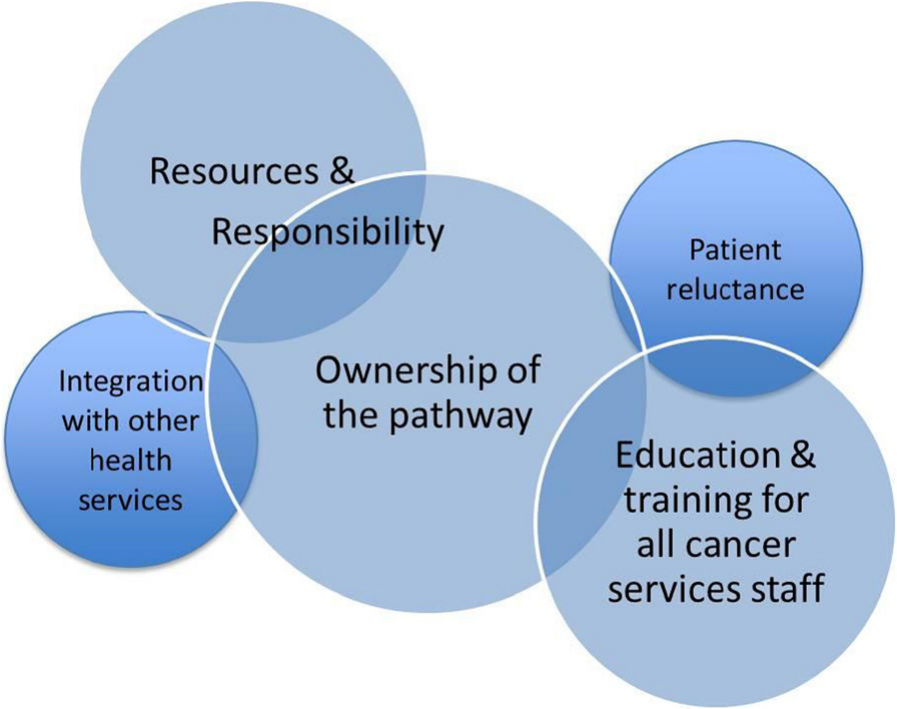
**Intersecting qualitative themes identified in the analysis.**

The thematic analysis revealed five themes about the implementation of the clinical pathway, including: ownership; resources and responsibilities; education and training; patient reluctance to access support; and integration with health services beyond oncology. Figure 1 was developed by the authors to demonstrate the interconnectedness and overlap of the themes as they emerged from analysis of the participants’ quotes.

***Theme A:*Ownership of the pathway** by the whole cancer services team was the central theme of the study, to enable the pathway to be acceptable to everyone participating in its delivery. Ownership at the team level was seen to foster the development of a whole team approach directed towards a common goal and ensuring that the pathway also reflected the team’s needs.

*“…people need to feel that this is an important priority, that they’re involved in shaping it, localizing it, customizing it, that it reflects what they can do and achieve, that they’re supported in it. …the pathway document (is) a starting point and getting people engaged in it.”* (Psychiatrist 1)

*“…getting engagement with psychosocial services and the nursing staff… is really important because the bottom line is that at the end of the day they’re going to implement it.”* (Nurse – metro setting)

Ownership by cancer services leadership and hospital management was thought to be essential (e.g. through IT systems, policy, role descriptions) to ensure the pathway was integrated into service delivery and that staff were supported in delivering, monitoring and evaluating this care.

*“There needs to be explicit support from the institution that spending time on these issues is time well spent. That it’s valued and supported, people are given time to do it, and that it is a priority…so that everyone’s committed to trying to make it work… It needs leadership, it needs major clinician leaders to be supportive otherwise the screening won’t occur…”* (Psychiatrist 1)

*“…(it’s the) local champions that always makes a difference.”* (Psychiatrist 2)

Integration into existing hospital systems and documentation in the medical record where members of the team could access this information was emphasised, so that screening results could be used in meaningful ways.

*“…support should be at the system level in terms of how it’s integrated, in routine documentation, in IT systems and in quality review.”* (Nurse clinician-researcher)

*“(we need the)…ability to track referrals and see whether the patient actually saw the psycho-oncologist because it doesn’t always happen… sometimes the referral gets lost or someone forgets to make a phone call, … and to have that in some sort of standardized, accessible way, ideally as part of the medical record.”* (Medical oncologist)

Participants also reflected on the advantages of having screening and pathway implementation incorporated into policy, either at the local hospital or jurisdictional level.

*“And if… you’ve got senior buy-in to say ‘this is an expectation of our cancer services… if you provide the support underneath that and the resourcing of the implementation to a certain degree, you’re kind of covering both ends.”* (Nurse – metro setting)

***Theme B: Resources and responsibility****for screening and after care* were viewed as two inter-related concepts. For the resources required to implement a clinical pathway, participants spoke about: a) the lack of staff time to administer a screening tool and interpret the results, and b) the lack of qualified psychosocial staff to provide after-care for distressed patients. Lack of staff meant that some participants expressed significant concerns about not being able to provide care to those with the greatest needs.

Participants reflected that from a ‘team’ perspective, having a clinical pathway could provide resources that would facilitate a more effective way of working together. Most participants considered having to renegotiate or make explicit the resources within the team to implement a pathway was not an insurmountable barrier, but an issue that requires careful planning and execution.

*“… as a team… how are we going to address it and can we address it, have we got the manpower to actually follow through with it? So implementing I think would take a bit of an effort but it’s not impossible.”* (Rural palliative care nurse)

*“…the two biggest problems are multiple points of entry to the system, throughout the local health district, and secondly is having the psycho-oncology manpower to follow up on referrals.”* (Social worker 1)

The concept of responsibility incorporates participant concerns about duty of care or ethical responsibility for acting to alleviate distress if identified during the screening process. Participants were particularly concerned that screening should not be introduced *unless* there was a clear clinical pathway and staff with designated responsibilities for each step of screening, interpreting results and ensuring after-care to ensure distressed patients received the after care they needed. Participants strongly emphasised that screening was a means to an end and in isolation was unlikely to result in improved patient outcomes.

*“handing somebody something like the distress thermometer and problem checklist is easy enough to do… it’s who then goes on to deal with the results from that?”* (Psychologist 1)

There was a strong sense of responsibility for those with greater levels of distress, and concern about the challenges of helping such patients, and the need for health professional expertise to provide appropriate interventions.

*“Some people do need more intense therapy to deal with the problems that they’re going through… I would like us to have psychiatric care available for… patients with very severe distress or the complex ones. … if I could get expert help with the diagnosis – I can manage with more confidence… With things like some of the behavioural therapies, you know, I don’t really have time to give people that advice, it’s good to have someone who can give that.”* (Palliative care physician)

Participants noted that staff roles were often unclear, and at times there were tensions around negotiating this within the team. Developing a shared team understanding of roles and responsibilities was considered an enabler to assist referrals made to psychosocial team members.

“… *I think that there needs to be some clarity around who’s responsible for overseeing it across the patient’s journey.”* (Nurse clinician-researcher)

*“(there is) …a lack of clarity about who’s role it is, who the decision maker is… It’s not that uncommon that someone says ‘well that’s my role’ and everyone in the rest of the team goes ‘is it?’”* (Nurse metro setting)

*“…it is not just the access to the consultation liaison services, or the psychosocial services, it’s actually also having health professionals who are aware of this as an issue and are prepared to refer.”* (Psychiatrist 2)

### Theme C: Education and training

Education was perceived as a core activity, to enable identification of staff roles and responsibilities and build ownership of the pathway. Key components of education programs included basic education about psychosocial issues in cancer, how to use screening tools and the clinical pathway for oncology health professionals, communication skills training for staff in dealing with distressed patients, as well as education for patients in the importance and availability of psychosocial care.

“*I think having everyone… involved in the education so there’s ownership from the institution or from the clinicians about it…”* (Social worker 2)

*“widespread education that highlights… the importance of psychosocial assessment, as well as the efficacy of psychosocial intervention… delivered in terms of… how it reduces clinician time by identifying and intervening with psychological distress in a timely manner, so you’re likely to reduce your face-to-face time with a clinician which they’ll always like… and … evidence about preventing unnecessary admissions and presentations, as well as improving quality of life and satisfaction with care.”* (Social worker 1)

Presenting an evidence base and educating clinicians about the benefits of screening for distress was also noted by the three physician participants. This included education for the whole team about the screening instrument, and the benefits of using it.

*“You’d probably need to up-skill more than one person so that if one person’s away other people could do it… education gets past a lot of these barriers.”* (Palliative care physician)

One psychiatrist clearly articulated this when s/he said: *“we focus a great deal on changing clinicians’ expectations and skills, but I don’t think we’ve even tackled too closely an understanding of what’s needed in order to make services more acceptable to patients.”*

### Theme D: Patient reluctance

Participants spoke about the challenge of patient reluctance to access psychosocial services; some patients are unaware of available services, others are resistant to seeking assistance because of stigma associated with mental illness. Participants recounted that some patients will accept a referral but not make an appointment, others value stoicism and presenting a ‘brave face’. Whilst patient reluctance was viewed as a significant barrier, participants thought it could be overcome by sensitive and careful referral.

*“For a lot of people, it’ll be the first time they’ve ever spoken to someone about distress, so it’s not a simple step… it needs to be done in a thoughtful and sensitive way.”* (Psychiatrist 1)

*“…there’s a great resistance with some people… (there’s) one gentleman at the moment who is just refusing point blank to see anybody, yet his wife is telling me that he’s spending most of his time in tears at home and… mentioning one of the children’s names he will just dissolve into tears, but we’re not actually seeing him…”* (Rural palliative care nurse)

*“…generally people don’t know what they’re entitled to, what’s available, what the resources are.”* (Social worker 1)

*“…there needs also to be a degree of attention to the stigma of mental health interventions among people with cancer. .. a fairly high percentage of people who decline services. We need some sort of educational promotion program… to indicate that mental health care is an integrated and important part of cancer care.”* (Psychiatrist 1)

Participants conveyed a sense of hopefulness that a clinical pathway would go some way to ameliorating patient reluctance. Thus, this theme intersects with the themes of education and training, which would include strategies to educate patients about the benefits of psychosocial services, and with that of ownership, where a whole team approach may work as an enabler to overcome patient reluctance.

### Theme E: Integration with health services beyond oncology

In order for a clinical pathway to be successfully implemented, participants considered there needs to be better integration and communication across health services including primary care, mental health, and the palliative care services. This was particularly relevant when considering the referral of acutely distressed patients or those with pre-existing conditions.

*“… a lot of GPs… would like a more active role in the acute treatment phase, better communication with the cancer team, and …. the opportunity to have some role in helping to support patients if they are suffering distress, …given that we usually have a pre-existing long-term relationship… and are well placed to be able to provide some additional support.”* (General practitioner)

With regards to mental health services,

*“Our mental health services are not all that good at responding to people with physical illness. For people who have pre-existing mental health problems who develop cancer, where you need good partnership between the systems, the mental health clinician needs to stay involved and care for that person.”* (Psychiatrist 1)

Similarly, with regards to palliative care services,

*“Sometimes it’s difficult to know how much I can attribute to cancer and how much is actually a psychiatric disorder. Aged care psychiatry could be a great help (but).. as soon as they see the word ‘cancer’ they just send them straight back (to palliative care) and that’s a real problem.”* (Palliative care physician)

### Acceptability of a clinical pathway

Several participants talked about how a clinical pathway could be acceptable in order to facilitate its implementation, including emphasising its benefits of supporting evidence. Others noted that health professionals’ fatigue from having to implement pathways in the clinical setting (‘pathway fatigue’) would need to be overcome for successful implementation.

*“Clinical pathways are used in lots of different areas and the ease at which it is to implement these things is a challenge and… (there is a) degree of fatigue around different things that get implemented… particularly once you get down to department level.”* (Nurse – metro setting)

*“I think the biggest problem is actually producing data that shows that this is beneficial, we know that what we do can make a difference, we know that we can’t squander scarce resources, we’ve just got to come up with data to support that …. otherwise you’re not going to get funding.”* (Psychiatrist 2)

*“Someone needs to show that this will actually lead to not necessarily a substantial increase in referrals to the high end of the services, but actually a better utilization of those resources.”* (Nurse – metro setting)*.*

Ultimately, participants in the study were generally supportive of the framework, despite raising concerns and describing potential barriers. As one psychologist said:

*“…everybody agrees that routine screening should be implemented (but nobody can decide how best to do it or when it should be done or who’s going to be responsible for it, or what you do with the information once you get it) so having a document or a framework like this is really useful.”* (Psychologist 1)

## Discussion

This qualitative paper identified five themes relating to barriers and enablers that are likely to be encountered during the implementation of a clinical pathway for anxiety and depression in cancer care. These intersecting themes incorporate ownership; resources and responsibility; education and training; patient reluctance; and integration with health services beyond oncology. This is the first study specific to a clinical pathway implementation in psychosocial oncology to formally document such themes using a qualitative approach.

Strategies for successful distress screening implementation in cancer are frequently based on review articles, commentary and lessons learned during attempts to implement screening in Canada, the US and UK [5,26,27]. Our research significantly enhances the evidence base, by identifying enabling strategies that integrate screening *and* after-care in cancer services. The identified themes are consistent with those documented about barriers and enablers to implementation of evidence into practice more generally [28]. The ownership of a clinical pathway by the whole cancer services team, including leadership, was shown to be crucial to successful implementation. The extant literature also confirms the importance of being strategic, collaborative and explicitly aware of the culture of health care delivery [5,29] and engaging clinicians in the implementation process [27].

Lack of resources [time, finances, equipment and skills] are well documented as significant barriers to guideline implementation in chronic health conditions [30-32], distress screening in cancer [33], and in organising psychosocial services [34]. Enabling strategies include using phased-in approaches to implementation, using research and evaluation as support tools and partnering proactively with stakeholders [29]. Our findings further suggest integrating clinical pathways into existing hospital systems including cancer services policy and IT systems, and for hospital leadership to endorse such activities. Participants emphasised the ability to track referrals and demonstrate outcomes to ensure high quality care and evaluate services.

The lack of education and training to accompany implementation was identified as critical and has been identified as a significant barrier to distress screening also [8]. Our study also revealed that education was considered to be significant enabler for successfully implementing a clinical pathway. Participants emphasised the need for cancer services staff to understand each other’s roles and responsibilities in order to integrate processes for screening, referral and providing after-care for patients.

Patient reluctance or refusal of treatment for distress is another well documented barrier to care [10,35-37]. Although fewer participants in this study reflected on patient reluctance, there was very strong support for education for patients as a means to reducing the stigma attached to mental health issues and referral. Openly acknowledging resistance from patients and staff can facilitate greater acceptance of distress screening and after-care [29].

This study contributes new findings about acknowledging services outside of oncology that patients may already utilise, particularly integration with mental health services and existing relationships with the general practitioner. To our knowledge, this aspect has not been previously discussed in the literature, and yet is clearly of considerable relevance.

### Interpreting barriers and enablers within identified themes

Barriers and enablers to implementation were evident across all five themes. We found it interesting that participants identified each theme as *both* a barrier and enabler to implementation. For example, the absence of education and training was perceived as a barrier, but when included as a core implementation strategy it was perceived as an enabler. Resources and responsibility were strongly interpreted as barriers if not addressed; yet improvements to organising resources were perceived as an enabler. Ownership by the team was initially perceived as a barrier, but as the interviews progressed, many participants became more positive in their views that an engaged team that was willing to shape and lead pathway implementation could work cooperatively to overcome known barriers. Participants clearly identified that demonstrating the *efficacy and efficiency* of an evidence-based pathway – in more accurately identifying anxiety and depression, reducing unplanned admissions and clinician time in consultations – would facilitate implementation.

The limitations of this study are considered to be the small sample size and using a purposive sample, although multi-disciplinary participant expertise and experience with the local health system is advantageous, and thematic saturation was achieved. We note that a larger sample would be useful in identifying whether barriers and enablers were perceived differently by medical, nursing and allied health professionals, as well as by patients. In order to address this limitation and building on the current results, a Delphi process with a larger sample has been undertaken to establish consensus about the draft clinical pathway from a wider stakeholder group; data analysis is in progress.

## Conclusions

Our results demonstrate consensus among multi-disciplinary experts that a draft clinical pathway has the potential to enable screening for anxiety and depression in cancer care that is not considered in isolation from after-care and actively responding to patients’ needs [8]. The five themes – when perceived as both barriers and enablers – provide a basis for a future implementation plan that includes strategies to overcome barriers and facilitate positive uptake. The next steps are to design and deliver this clinical pathway, addressing the context of each health service, to ensure team ownership, endorsement by leaders, education and training strategies for both patients and health professionals, and the integration of the pathway into existing cancer services.
